# The prevalence, incidence, and admission rate of diagnosed schizophrenia spectrum disorders in Korea, 2008–2017: A nationwide population-based study using claims big data analysis

**DOI:** 10.1371/journal.pone.0256221

**Published:** 2021-08-12

**Authors:** Yoon-Sun Jung, Young-Eun Kim, Dun-Sol Go, Seok-Jun Yoon

**Affiliations:** 1 Department of Public Health, Graduate School, Korea University, Seoul, Korea; 2 Department of Big Data Strategy, National Health Insurance Service, Wonju, Korea; 3 Department of Health Care Policy Research, Korea Institute for Health and Social Affairs, Sejong, Korea; 4 Department of Preventive Medicine, Korea University College of Medicine, Seoul, South Korea; Rutland Regional Medical Center, UNITED STATES

## Abstract

This study estimated the prevalence and incidence rate of schizophrenia, schizotypal, and delusional disorders (SSDD) in Korea from 2008 to 2017 and analyzed the hospital admission rate, re-admission rate, and hospitalization period. It used the Korean nationwide National Health Insurance Service claims database. SSDD patients who had at least one visit to Korea’s primary, secondary, or tertiary referral hospitals with a diagnosis of SSDD, according to the International Classification of Diseases, 10th Revision (ICD-10), were identified as SSDD cases if coded as F20-F29. Data were analyzed using frequency statistics. Results showed that the 12-month prevalence rate of SSDD increased steadily from 0.40% in 2008 to 0.45% in 2017. Analysis of the three-year cumulative prevalence rate of SSDD showed an increase from 0.51% in 2011 to 0.54% in 2017. In 2017, the five-year cumulative prevalence rate was 0.61%, and the 10-year cumulative prevalence rate was 0.75%. The hospital admission rate among SSDD patients decreased from 2008 (30.04%) to 2017 (28.53%). The incidence of SSDD was 0.05% and no yearly change was observed. The proportion of SSDD inpatients whose first hospital visit resulted in immediate hospitalization was 22.4% in 2017. Epidemiological indicators such as prevalence, incidence, and hospitalization rate play an important role in planning social and financial resource allocation. Therefore, efforts to produce more accurate epidemiological indicators are very important and this study’s findings could have a significant social impact.

## 1. Introduction

Mental illness has a significant impact on individuals, families, society, health care systems, and the economy. It also garners considerable national interest due to the occurrence of numerous related social problems. Particularly, severe mental illnesses, such as schizophrenia, are a public health problem which can cause a great deal of personal and social harm and require continuous treatment strategies and public systems to prevent recurrence following acute intensive care. Given that social investment is necessary for the treatment and rehabilitation of these illnesses, it is important to understand the magnitude of mental illness’ impact by examining the prevalence, incidence, and hospitalization rate.

Research has been conducted globally to ascertain the prevalence of mental illness. For example, the World Health Organization (WHO) conducts “The WHO World Mental Health Surveys” in all WHO countries to obtain cross-national information on the prevalence rate of and the correlation between mental illness, drug use, and behavioral disorders. The survey examines 29 countries in six regions (Africa, America, the Middle East, Europe, Southeast Asia, and the Western Pacific) using the Composite International Diagnostic Interview (CIDI) survey instrument [1]. However, this survey does not include severe mental illnesses, such as schizophrenia. In Korea, a catchment area study has been conducted every five years since 2001, using the Korean version of the CIDI instrument to establish the prevalence of mental illness [2]. However, the epidemiologic indicators of severe mental illness (e.g., schizophrenia, bipolar affective disorder), with low prevalence or high hospital or facility admission rate, are not surveyed. Severe mental illnesses with low prevalence tend to have a long illness duration, high symptom severity, and require significant mental health resources; it is essential to understand the size of the problem.

This study aimed to estimate the prevalence and incidence rate of schizophrenia, schizotypal, and delusional disorders (SSDD) in Korea from 2008–2017, and to analyze the hospital admission rate, re-admission rate, and hospitalization period.

## 2. Materials and methods

### 2.1 Dataset

The claims data from National Health Insurance Service (NHIS) of Korea was used as the primary source of data. The NHIS of Korea covers almost the entire population of Korea and constructs the nationwide claims database [3]. As of 2019, it includes not only complete medical records of about 53 million people (the National Health Insurance covers 97.2% of the population, while the remaining 2.8% are covered by the Medical Aid program), but also the eligibility database, the national health screening database, and the health care utilization database. The health care utilization database includes information on inpatient and outpatient service usage. In this study, we used de-identified data from the NHIS claims database, which holds healthcare utilization information on hospital inpatients and outpatients, including patient demographics, date of admission and discharge, date of visit, principle diagnosis, and complications based on the International Classification of Diseases, 10^th^ edition (ICD-10).

SSDD patients who had at least one visit to Korea’s primary, secondary, or tertiary referral hospitals with a diagnosis of SSDD, as determined by the ICD-10 codes F20-F29, were identified as SSDD cases. These cases included Schizophrenia (F20), Schizotypal disorder (F21), Persistent delusional disorders (F22), Acute and transient psychotic disorders (F23), Induced delusional disorder (F24), Schizoaffective disorders (F25), Other nonorganic psychotic disorders (F28), and Unspecified nonorganic psychosis (F29). The database used in this study included all hospitalizations and outpatient visits due to SSDD.

### 2.2 Statistical analyses

We used frequency measures to calculate prevalence, incidence, and hospitalization rates. The 12-month prevalence rate of SSDD was calculated as the number of people who were diagnosed with SSDD divided by the number of people that received National Health Insurance (NHI) coverage that year. In addition, the cumulative prevalence of three, five, and ten years were calculated based on each year’s prevalence rates. For example, the three-year cumulative prevalent cases in 2017 were defined as people who visited a medical institution after being diagnosed with SSDD from 2015 to 2017.

The hospital admission rate was calculated as the number of inpatients due to SSDD among the prevalent cases. The re-admission rate was defined as the percentage of patients who were re-admitted among discharged patients each year. In this case, re-admission within one day was considered as the same hospitalization event. In addition, the average length of in-hospital stay after admission was calculated.

To calculate the incidence rate, we excluded those who had at least one visit to a hospital or clinic due to SSDD in the previous five years. We also calculated the proportion of patients among incident cases who were hospitalized for their first medical intervention due to SSDD. All calculations were performed using SAS 9.4 (SAS Institute, Cary, NC, USA).

### 2.3 Ethics approval and consent to participate

This study was approved by the National Health Insurance Service of Korea (No. NHIS-2019-1-378) and the Institutional Review Board (IRB) of Korea University (IRB No. KUIRB-2019-0203-01). Informed consent was waived by the board.

## 3. Results

The 12-month prevalence rate of SSDD increased steadily from 0.40% in 2008 to 0.45% in 2017. The analysis of the 12-month prevalence rate of SSDD by gender indicates that the prevalence for men was higher than that for women before 2012; this trend has reversed since 2012. In 2012, men and women had a similar prevalence rate of 0.42%, but over time, the gap between the genders widened. In 2017, the prevalence rate for women was 0.46%, while being 0.43% for men. The prevalence rate of SSDD by age group showed the highest prevalence among the 40–59 years age group. The prevalence rate of SSDD tended to decrease steadily among the 30–39 year-olds, and stagnate or decreased slightly among the 40–49 year-olds. Conversely, the prevalence rate of SSDD for all age groups above 50 tended to increase, with the fastest increase among 60–69 year-olds. The prevalence rate of SSDD varied substantially according to the type of medical insurance. For NHI beneficiaries, the prevalence rate increased by 0.03% over the decade, from 0.25% in 2008 to 0.28% in 2017. However, the prevalence rate of medical aid beneficiaries increased by 1.88% over ten years, from 4.16% in 2008 to 6.04% in 2017. Thus, the prevalence rate of SSDD for medical aid beneficiaries in 2008 was 16.6 times higher than that of NHI beneficiaries; this difference gradually increased to 21.3 times in 2017. The prevalence rates of Schizophrenia (F20) and Schizoaffective disorders (F25) were 0.36% and 0.03%, respectively, in 2017. In 2017, 79.89% of SSDD patients were diagnosed with Schizophrenia (F20) (Table 1).

**Table 1:** 12-month prevalence rate of schizophrenia, schizotypal and delusional disorders, 2008–2017.

	2008	2009	2010	2011	2012	2013	2014	2015	2016	2017
**12-month period Prevalence rate**	0.40	0.41	0.40	0.41	0.42	0.43	0.43	0.44	0.44	0.45
**Gender**										
Men	0.40	0.41	0.40	0.41	0.42	0.42	0.42	0.43	0.43	0.43
Women	0.39	0.40	0.39	0.40	0.42	0.43	0.43	0.44	0.45	0.46
**Age, year**										
0–9	0.00	0.00	0.00	0.00	0.00	0.00	0.00	0.00	0.00	0.00
10–19	0.07	0.07	0.06	0.06	0.06	0.06	0.06	0.06	0.07	0.07
20–29	0.29	0.29	0.27	0.27	0.28	0.28	0.28	0.28	0.29	0.29
30–39	0.55	0.53	0.50	0.48	0.47	0.46	0.45	0.44	0.44	0.44
40–49	0.68	0.69	0.69	0.70	0.71	0.70	0.69	0.69	0.68	0.67
50–59	0.60	0.62	0.63	0.65	0.67	0.68	0.69	0.70	0.71	0.72
60–69	0.43	0.45	0.45	0.47	0.51	0.53	0.55	0.57	0.58	0.60
70–79	0.34	0.34	0.32	0.34	0.37	0.39	0.39	0.41	0.42	0.42
80+	0.38	0.38	0.35	0.36	0.42	0.43	0.42	0.45	0.45	0.46
**Medical coverage type**										
National health insurance	0.25	0.25	0.25	0.25	0.27	0.27	0.27	0.28	0.28	0.28
Medical aid	4.16	4.78	4.66	5.03	5.43	5.67	5.79	5.52	5.82	6.04
**Specific ICD-10 code**										
Schizophrenia (F20)	0.33	0.33	0.33	0.34	0.34	0.35	0.35	0.35	0.35	0.36
Schizotypal disorder (F21)	0.00	0.00	0.00	0.00	0.00	0.00	0.00	0.00	0.00	0.00
Persistent delusional disorders (F22)	0.02	0.02	0.02	0.02	0.02	0.02	0.02	0.02	0.02	0.02
Acute and transient psychotic disorders (F23)	0.01	0.01	0.01	0.01	0.01	0.01	0.01	0.01	0.01	0.01
Induced delusional disorder (F24)	0.00	0.00	0.00	0.00	0.00	0.00	0.00	0.00	0.00	0.00
Schizoaffective disorders (F25)	0.02	0.02	0.02	0.02	0.03	0.03	0.03	0.03	0.03	0.03
Other nonorganic psychotic disorders (F28)	0.01	0.01	0.01	0.01	0.01	0.01	0.01	0.01	0.01	0.01
Unspecified nonorganic psychosis (F29)	0.03	0.03	0.03	0.03	0.04	0.04	0.04	0.04	0.04	0.04

Note. Unit for all figures is percentage.

Analysis of the three-year cumulative prevalence rate of SSDD showed an increase of 0.03% from 2011 to 2017, while the five-year and 10-year cumulative prevalence rate remained relatively constant with a 0.01%, and 0% increase, respectively (Table 2).

**Table 2:** 12-month prevalence rate of schizophrenia, schizotypal and delusional disorders, 2008–2017.

	2011	2012	2013	2014	2015	2016	2017
1 year prevalence rate	0.41	0.42	0.43	0.43	0.44	0.44	0.45
3 year cumulative prevalence rate	0.51	0.51	0.52	0.52	0.53	0.53	0.54
5 year cumulative prevalence rate	0.60	0.60	0.59	0.59	0.60	0.60	0.61
10 year cumulative prevalence rate	0.75	0.76	0.76	0.76	0.76	0.76	0.75

Note. Unit for all figures is percentage.

The hospital admission rate among SSDD patients decreased from 2008 (30.04%) to 2017 (28.53%). Analysis by gender indicates that the hospitalization rate for men was higher than that for women; in 2017, the hospitalization rate was 32.02% for men and 25.00% for women. Analysis by medical coverage type shows that the hospitalization rate of medical aid beneficiaries was 22.75% higher than that of NHI beneficiaries. The re-admission rates for patients with SSDD discharged from the hospital each year increased gradually from 40.23% in 2008 to 48.98% in 2017. The occurrence of re-admission of discharged patients with SSDD was 10.11% within three days after the previous discharge event, 18.50% after seven days, 26.09% after 14 days, and 33.14% after 30 days. The re-admission rate was 41.03% within three months and 46.84% within six months after the previous discharge event (Table 3).

**Table 3:** Admission and re-admission rate of schizophrenia, schizotypal and delusional disorders, 2008–2017.

	2008	2009	2010	2011	2012	2013	2014	2015	2016	2017
**Admission rate**	30.04	29.91	30.85	30.81	30.38	29.87	29.54	29.14	28.38	28.53
**Gender**										
Men	32.51	32.51	33.26	33.43	33.39	33.08	32.83	32.30	31.50	32.02
Women	27.27	26.97	27.64	27.56	26.88	26.27	25.98	25.78	25.11	25.00
**Medical coverage type**										
National health insurance	20.99	20.62	22.61	22.63	22.28	21.79	21.41	21.09	20.22	19.69
Medical aid	44.08	43.74	42.85	42.74	42.85	42.51	42.53	42.07	41.25	42.44
**Re-admission rate (among discharge patients)**	40.23	41.08	42.25	42.28	47.46	44.13	44.22	44.70	45.55	48.98
within 3 days after discharge	6.61	6.27	7.38	7.59	7.70	8.51	8.77	9.02	9.57	10.11
within 7 days after discharge	12.90	13.26	14.88	14.91	14.37	15.77	16.16	16.35	17.35	18.50
within 14 days after discharge	18.25	18.76	21.01	21.18	20.14	22.24	22.61	22.98	23.85	26.09
within 30 days after discharge	23.57	24.18	26.54	27.06	25.82	28.35	29.08	29.43	30.89	33.14
within 60 days after discharge	30.56	31.35	32.02	32.25	38.37	34.17	34.68	35.14	36.26	38.46
within 90 days after discharge	33.42	34.27	34.77	34.96	40.93	37.16	37.34	37.85	38.87	41.03
within 180 days after discharge	37.82	38.64	39.52	39.70	45.02	41.72	41.75	42.26	43.20	46.84

Note. Unit for all figures is percentage.

**Fig 1:**
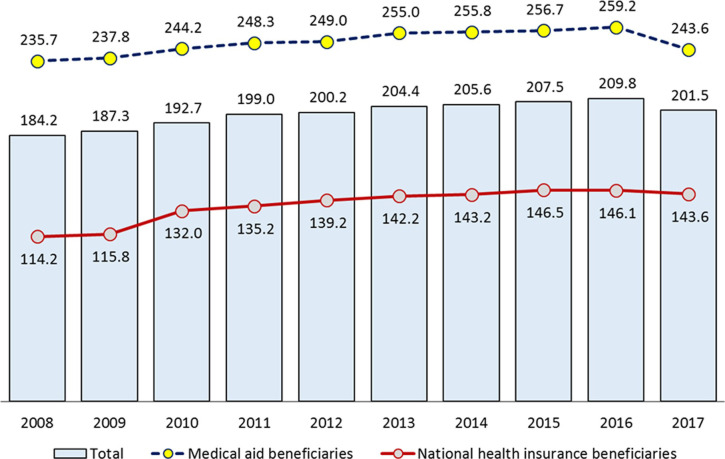
Length of hospital stay for schizophrenia, schizotypal and delusional disorders by medical coverage type, 2008–2017. (Unit: days).

The analysis of average length of stay for SSDD inpatients for each year showed an increase of approximately 17.4 days, from 184.2 days in 2008 to 201.5 days in 2017. The length of hospital stay differed according to the type of medical insurance. In 2017, the average length of hospital stay for NHI beneficiaries was 143.6 days and that for medical aid beneficiaries was 243.6 days (Fig 1).

The incidence rate of SSDD was 0.05%, and no change was observed across each year. The incidence rate in women presenting with SSDD was slightly higher than women, but the size of the difference was very small (0.01%). In contrast, the incidence rate by medical coverage type differed considerably. The incidence of SSDD in NHI beneficiaries was 0.04%, compared with 0.36% in medical aid beneficiaries. The proportion of inpatients, whose first use of health services for SSDD resulted in hospitalization (admitted immediately without outpatient service use), accounted for 22.4% in 2017 (Table 4).

**Table 4:** Incidence rate and admission rate for first use of health services for schizophrenia, schizotypal and delusional disorders, 2008–2017.

	2008	2009	2010	2011	2012	2013	2014	2015	2016	2017
**Incidence rate**	0.06	0.06	0.05	0.05	0.05	0.05	0.05	0.05	0.05	0.05
**Gender**										
Men	0.06	0.05	0.05	0.05	0.05	0.05	0.05	0.05	0.05	0.05
Women	0.07	0.06	0.05	0.05	0.06	0.05	0.05	0.05	0.06	0.06
**Medical coverage type**										
National health insurance	0.05	0.05	0.04	0.04	0.05	0.04	0.04	0.04	0.04	0.04
Medical aid	0.40	0.37	0.30	0.34	0.32	0.32	0.29	0.29	0.30	0.36
**Proportion of inpatients whose first use of SSDD is hospitalization (among incidence case)**	24.5	26.9	31.0	28.3	26.1	26.4	26.2	24.8	23.5	22.4

Note. Unit for all figures is percentage.

## 4. Discussion

This study aimed to provide accurate estimates of epidemiological indicators such as the prevalence, incidence, and hospitalization rates of SSDD, one of the most common severe mental illnesses, in Korea. This study is also the first to calculate the epidemiological indexes for SSDD using NHIS big data claims of all Koreans. Korea has a national health insurance system operated for the entire population by a single insurer. Thus, the NHIS big data claim information, covering a population of about 53 million people, is an optimal database for calculating treatment prevalence rate.

In Korea, a survey on the prevalence of mental illness is conducted every five years for adults living in the community. Given that this survey involves only adults living in the community, it is difficult to determine the prevalence rate of severe mental illnesses such as schizophrenia. In fact, the 2016 mental illness survey was conducted on only 5,102 people, and the annual prevalence rate was estimated using data from the 2015 Population and Housing Census as the standard population. As of 2016, the number of patients residing in the community with schizophrenia spectrum disorders (including schizophrenia, schizophreniform disorder, schizoaffective disorder, delusional disorder, and brief psychotic disorder) was estimated to be 63,361 (0.20%), and the annual prevalence rate was estimated to be 0.28% (n = 113,850) when adjusted for the number of patients in hospitals, mental health care facilities, and homeless shelters [2]. However, when considering the survey findings, it is likely that the prevalence of schizophrenia spectrum disorders may have been underestimated for a number of reasons. First, schizophrenia spectrum disorder is one of the most severe mental disorders due to the severity and chronic nature of symptoms. Thus, despite having a schizophrenia spectrum disorder diagnosis or experiencing symptoms, the prevalence rate may be underestimated because patients with SSDD often hide their symptoms. Second, the Korean version of CIDI requires the cognitive ability to understand questions correctly and respond appropriately. Schizophrenia spectrum disorders are often accompanied by impaired cognitive function and thinking disorders, which may lead to individuals with SSDD not being able to answer the questions adequately [2]. This is supported by O’Connor and Parslow [4], who raised the issue that the CIDI tool is composed of complex and structured questions, and thus has the possibility of underestimating rates of prevalence of mental disorder in older adults. Because the questions included in CIDI require analysis of multiple symptoms, time-frames, and attributions, their explanation is that the application of CIDI is limited for older adults having poor cognitive skills; it involves tasks that require the rapid manipulation of large quantities of complex and new information. Given this situation, it is likely that the prevalence rate of schizophrenia spectrum disorders in the community was underestimated. In addition, patients with schizophrenia spectrum disorders often stay in facilities; community surveys alone are not enough to estimate their prevalence. For this reason, estimating prevalence rate from health insurance claims data has more policy and academic significance than estimation through large-scale surveys. The 2016 prevalence rate of SSDD in this study was 0.44%, which is higher than the 0.28% calculated by the Survey of Mental Disorders in Korea in 2016. Nevertheless, concerns of underestimation remain, because claims data do not include the estimate for untreated or undiagnosed patients.

According to the National Institute of Mental Health, the prevalence rate of schizophrenia in the United States, derived from household-based surveys, interviews, and medical records, is estimated to range from 0.25% to 0.64% [5–7]. Further, Goldner et al. [8] pooled data from 18 international studies and estimated the global prevalence rate of schizophrenia at 0.34%. In comparison, Saha et al. [9], identified 132 core studies, 15 migrant studies, and 41 studies based on other special groups and estimated a median (10th to 90th percentile) of 3.3 (1.3–8.2) per 1000 persons for the distributions per period (i.e. 1 month to 1 year). Therefore, the prevalence rate of 0.40% to 0.45% calculated in this study is considered reasonable when considering the standard of international research. It is noteworthy that there was an increase in the prevalence rate of SSDD among adults who are 50 years and older. In particular, the prevalence rate among those in their 60s increased most rapidly between 2008 and 2017. In relation to this, Cho et al. [10] pointed out the problem with the timing of treatment for SSDD; the delay in treatment time was one of the reasons for the increase in the prevalence rate of SSDD in older adults. Young SSDD patients are not treated in a timely manner and receive treatment only at older ages. This can explain the higher prevalence rate of SSDD among older adults, rather than the possibility that schizophrenia develops in old age. This is more convincing when compared with trends in other age groups. It is known that the psychotic features of schizophrenia typically emerge between the late teens and mid-30s [11]. Nevertheless, the prevalence rate in the age group under 30 remains stagnant for 10 years and the prevalence rate in the 30s decreases by 0.11%p. Considering these results comprehensively, it can be inferred that the treatment of young SSDD patients is being delayed, and that an environment in which patients who are at high risk of developing SSDD can receive timely treatment is not being created.

In this study, while the incidence rate of SSDD remained constant over time, the prevalence rate increased. It is inferred that the number of patients who visit the hospital increased as the awareness of the necessity of treatment increased, rather than the actual number of patients. Moreover, the prevalence rate in women was higher than that in men. In general, the prevalence rate of SSDD is not known to vary by gender; this result is therefore inconsistent with studies in other countries, such as Thailand and the United States [7, 12, 13]. Nevertheless, the higher prevalence in women suggests that women are more likely to receive treatment than men in Korea, and conversely, men are more likely to discontinue treatment or not receive treatment. Therefore, intervention and social investment are required for untreated patients.

The prevalence rate of SSDD in the 40–60 age group was higher than in other age groups. Given that these age groups are associated with the highest productivity period, the social losses are also be expected to be significant. According to Lee et al. [14], who studied the economic burden of disease in Korea, the economic burden of schizophrenia is ranked 17^th^, between diabetes mellitus and lung cancer, out of 260 diseases. Intervention studies on how to manage SSDD illnesses are needed in these high productivity age groups.

In this study, to determine the difference of SSDD prevalence rate based on income, we used the type of medical coverage as a proxy indicator of income. The prevalence rate among medical aid beneficiaries with a relatively lower income was 6.04% in 2017, which was approximately 21.3 times higher than that of NHI beneficiaries (0.28%), and had increased over the years since 2008. In addition, the average length of hospital stay for medical aid beneficiaries was 100 days longer than that for NHI beneficiaries, showing a difference by income level. It was observed that the high prevalence of SSDD among medical aid beneficiaries was related to a decrease in income level after the onset of the disease. Nevertheless, further research is needed on the underlying cause of the 21.3-fold difference between the income levels.

Globally, mental health paradigms are shifting from hospitalization to community treatment, and mental health professionals also talk about the importance of community treatment. Per global trends, the Organization for Economic Co-operation and Development (OECD) has recommended a transition from inpatient-based care to community-based care [15]. Accordingly, efforts have been made in Korea to switch from hospitalization in mental health facilities to community treatment for patients with severe mental illnesses. The Mental Health and Welfare Act was revised on May 30, 2017, to dramatically strengthen human rights protections, improve mental illness anti-discrimination policies, and provide welfare services based on the improvement of the compulsory hospitalization system. Particularly, the standards and procedures for hospitalization by the person with protective obligation (compulsory admission and involuntary admission) were significantly strengthened. However, recent instances of violent crimes committed by patients with schizophrenia have been indiscriminately delivered to the public by the media without considering how these reports would impact the stigma surrounding patients with schizophrenia. Through numerous media reports, it has been emphasized that the person who committed the crime was an SSDD patient [16], and only seeing the characteristic of the violent individual as being an SSDD patient can lead the public to recognize patients with SSDD as ‘potential criminals’ [17]. However, according to Crime Analysis report by the Supreme Prosecutor’s Office in 2011, the crime rate for non-psychiatric patients was 1.2%, while the crime rate for people with mental illness was 0.08%. Thus, the probability of a person with mental illness committing a crime is only one-fifteenth that of a person without a mental illness diagnosis committing a crime. In addition, most of the crimes involving people with mental illness occurred before they received treatment; after receiving treatment, the risk of crime was reduced by more than 94% [18]. Media reports are socially expanding unconscious prejudices against patients with SSDD, and if such an atmosphere prevails in society, patients who actually need treatment may hide their symptoms and miss the opportunity to receive timely treatment, which can further intensify their problems. In other words, negative perceptions of severe mental illness have resulted in patients missing intensive care periods during the early stages of the illness, thus making the sustainability of treatment difficult. In addition, when patients with severe mental illness are discharged from the hospital, they are notified of the mental health welfare center in the community if they agree to further community-based treatment. However, community-based treatment is difficult because the center linkage process is not always smooth, such as when the patient does not agree to receive further community-based treatment. Considering this situation, the study’s results, which showed that the re-admission rate after discharge gradually increased and that one out of three discharged patients were re-admitted within a month, signify that the social support system for SSDD patients in the community is unstable. In other words, they show that the problem of repeated discharge and subsequent re-admission continues because the link between hospital and community treatment and adaptation of support services for continuous treatment management of SSDD patients is not ready.

The length of hospital stay for SSDD inpatients has increased steadily from 2008 to 2016, but this trend decreased in 2017. Particularly, for medical aid beneficiaries, the length of stay in hospitals per year has decreased and this can be seen as a positive effect of the revision of the Mental Health and Welfare Act. Nevertheless, long-term hospitalization and continuous re-hospitalization of patients with severe mental illness remain a major problem in Korea. This is evident when compared with OECD countries; the average length of stay in hospitals for schizophrenia patients in Korea is 237.8 days, which is nearly five times longer than the OECD average of 48.9 days [19]. While many other countries are moving towards deinstitutionalization, reducing the number of hospital beds and shifting to community care and residence, in Korea, long-term hospitalization and facility-oriented care still dominate. A multi-faceted approach is needed to improve long-term hospitalization, including the expansion of community care services.

The findings of this study indicate that 24.5% (6,046 patients) of SSDD incidence cases experienced immediate hospitalization without outpatient service use. This rate decreased since 2010 and can be interpreted as more prudent decision-making taking place when considering hospitalization of patients with severe mental illness. In other words, unnecessary initial hospitalization is decreasing. Despite these positive interpretations, one in five SSDD onset patients are still starting treatment with severe symptoms that require immediate hospitalization. In other words, about 6,000 SSDD patients experience worsening symptoms in the community without treatment. To reduce the number of patients in this scenario, research on their characteristics and social systems to induce early treatment is needed.

This study estimated the prevalence rate of SSDD using claims data, but the overall prevalence rate and prevalence rate by sex and age group may still be underestimated because the data only includes claims from patients with SSDD who visit or receive medical treatment. However, previous community surveys show that the majority of patients with schizophrenia are being treated [20]. Conversely, the results of this study based on big data may contain the possibility of overestimation. This is because some individuals could have a misdiagnosis of SSDD. Additionally, claims code errors could have occurred [21]; however, Wu et al.’s [7] sensitivity analysis established that this error was found in less than 5% of SSDD cases. Therefore, the big data-based prevalence estimation methodology used in this study may have led to a slight underestimation or overestimation, but its impact is unlikely to be significant.

## 5. Conclusions

The annual prevalence of SSDD using NHIS database was higher than the previous prevalence rate obtained from the Survey of Mental Disorders in Korea. In older adults, the prevalence has increased significantly over the past decade, while the prevalence in the 10–30 age group has been stagnant or decreased. These findings provide information on the prevalence of SSDD and medical utilization based on better estimates and can be used as evidence to develop the management strategies and policies to support patients at the national level. There is need to support people with high risk of developing SSDD to be diagnosed and received timely treatment. Strict regulations are needed to limit the use of stigmatizing language by media persons, and a socially inclusive approach need to be pursued so that people get a timely diagnosis and treatment without hiding their symptoms.
